# Expression and Purification of Recombinant GHK Tripeptides Are Able to Protect against Acute Cardiotoxicity from Exposure to Waterborne-Copper in Zebrafish

**DOI:** 10.3390/biom10091202

**Published:** 2020-08-19

**Authors:** Chung-Der Hsiao, Hsin-Hui Wu, Nemi Malhotra, Yen-Ching Liu, Ying-Hsuan Wu, Yu-Nung Lin, Ferry Saputra, Fiorency Santoso, Kelvin H.-C. Chen

**Affiliations:** 1 Department of Bioscience Technology, Chung Yuan Christian University, Chung-Li 320314, Taiwan; cdhsiao@cycu.edu.tw (C.-D.H.); nemi.malhotra@gmail.com (N.M.); d01b41008@ntu.edu.tw (Y.-N.L.); ferrysaputratj@gmail.com (F.S.); fiorency_santoso@yahoo.co.id (F.S.); 2 Master Program of Nanotechnology, Chung Yuan Christian University, Chung-Li 320314, Taiwan; 3 Department of Applied Chemistry, National Pingtung University, Pingtung 900391, Taiwan; johnson.hsinhui.wu@gmail.com (H.-H.W.); kitty101298@gmail.com (Y.-C.L.); koala8192@gmail.com (Y.-H.W.); 4 Department of Biomedical Engineering, Chung Yuan Christian University, Chung-Li 320314, Taiwan

**Keywords:** zebrafish, GHK tripeptide, cardiotoxicity, copper, protein expression

## Abstract

In this study, an alternative method is developed to replace chemical synthesis to produce glycyl-histidyl-lysine (GHK) tripeptides with a bacterial fermentation system. The target GHK tripeptides are cloned into expression plasmids carrying histidine-glutathione-S-transferase (GST) double tags and TEV (tobacco etch virus) cleavage sites at the N-terminus. After overexpression in *Escherichia coli* (*E. coli*) BL21 cells, the recombinant proteins are purified and recovered by high-pressure liquid chromatography (HPLC). UV-vis absorption spectroscopy was used to investigate the chemical and biological properties of the recombinant GHK tripeptides. The results demonstrated that one recombinant GHK tripeptide can bind one copper ion to form a GHK-Cu complex with high affinity, and the recombinant GHK peptide to copper ion ratio is 1:1. X-ray absorption near-edge spectroscopy (XANES) of the copper ions indicated that the oxidation state of copper in the recombinant GHK-Cu complexes here was Cu(II). All of the optical spectrum evidence suggests that the recombinant GHK tripeptide appears to possess the same biophysical and biochemical features as the GHK tripeptide isolated from human plasma. Due to the high binding affinity of GHK tripeptides to copper ions, we used zebrafish as an in vivo model to elucidate whether recombinant GHK tripeptides possess detoxification potential against the cardiotoxicity raised by waterborne Cu(II) exposure. Here, exposure to Cu(II) induced bradycardia and heartbeat irregularity in zebrafish larvae; however, the administration of GHK tripeptides could rescue those experiencing cardiotoxicity, even at the lowest concentration of 1 nM, where the GHK-Cu complex minimized CuSO_4_-induced cardiotoxicity effects at a GHK:Cu ratio of 1:10. On the other hand, copper and the combination with the GHK tripeptide did not significantly alter other cardiovascular parameters, including stroke volume, ejection fraction, and fractional shortening. Meanwhile, the heart rate and cardiac output were boosted after exposure with 1 nM of GHK peptides. In this study, recombinant GHK tripeptide expression was performed, along with purification and chemical property characterization, which revealed a potent cardiotoxicity protection function in vivo with zebrafish for the first time.

## 1. Introduction

### 1.1. GHK Tripeptide and Its Applications

GHK is a tripeptide with the amino acid sequence glycyl-l-histidyl-l-lysine. It has been observed to occur naturally in human saliva, plasma, and urine [1,2]. In human plasma, the level of GHK is typically 200 ng/mL at the age of 20, but it declines to 80 ng/mL by the age of 60 [1]. The naturally occurring tripeptide GHK was first described as a liver cell growth factor [3,4,5]. Later, as research continued, it was demonstrated to stimulate growth and differentiation of a number of cell lines [6,7,8,9,10] and act as a growth inhibitor for some cultured cells [11], establishing itself as a modulating peptide [12]. In several previous studies, the GHK tripeptide has also demonstrated its importance in biological activities such as angiogenesis [13,14], wound healing [15,16], bone repair [17], collagen synthesis [18], and is used in skincare products [19,20]. Besides its function as a potent antioxidant, GHK also possesses anti-inflammatory and regenerative properties and supports and promotes stem cell function and the synthesis of neurotrophic factors [21,22]. In some recent studies, GHK has demonstrated its efficiency in regulating a broad number of human genes at a very low concentration. At 1 μM, it was able to suppress 70% of genes overexpressed in metastatic colon cancer [21]. Moreover, recent developments in the genetic regulation of the GHK tripeptide suggest that it may belong to a class of epigenetic modifiers, exhibiting a broad range of protective and restorative properties [21]. Thus, the GHK tripeptide, as a modulator for cellular growth, can play a versatile role in the biomedical and biotechnological development of new drugs, with potential applications [12].

### 1.2. The Toxicity of Copper

Copper is an essential transition metal that can exist in the reduced Cu(0) and oxidized Cu(I) and Cu(II) states, allowing it to contribute to catalytic chemistry as a cofactor for enzymes and molecules inside the body. The existence of Cu in the tissues of marine animals has been recognized and known for a long time, and it has been established as an environmental pollutant that is harmful to humans as well as aquatic organisms [23,24,25,26]. Copper was detected in the blood of *Eledone* and *Helix pomatia* by Harless (1847), which demonstrated that it exists not as a free salt but in combination with proteins.

Despite its importance for the maintenance and regulation of nerve cells, red blood cells, and the immune system, copper in a toxic form might serve as a stress agent for fish, inhibiting several biological functions and causing some histopathological alterations [27]. Moreover, a high concentration of copper was found to be toxic in both fishes and humans [28]. In concordance with this statement, Monteiro et al. (2012) also mentioned that copper toxicity might disrupt biochemical functions and cellular morphology [29]. In another study by Heath et al. (1987), the majority of copper was located in the gills, kidney, liver, and skeletal muscle of fish [30]. An imbalance of copper metabolization may prove to be harmful, potentially leading to copper accumulation in the body. In addition, the imbalance of copper in the body has been found to be related to genetic disorders such as Menkes and Alzheimer’s. Thus, copper toxicity and deficiency have since been studied under various pathological conditions [31].

Cardiotoxicity is a condition where heart dysfunction or muscle damage has occurred, and it can lead to the disruption of blood flow in the heart, damage to cardiac muscles, heart rate irregularity, and even death. Furthermore, since it induces damage to the heart, electrophysiological abnormalities, as well as vascular atherosclerosis, are generated due to reactive oxygen species (ROS) and inflammation [32,33]. In a previous study, the effect of copper on cardiovascular function was reported in zebrafish. Copper treatment resulted in evident necrosis and the absence of a heartbeat after 24 h of incubation [34]. Furthermore, another study in zebrafish also found that exposure to concentrated copper (11–1000 µg/L) led to faster heart rates at 28 h postfertilization (hpf) zebrafish embryos, suggesting a stress response in fish embryos [35]. In addition, in invertebrate animals such as *Daphnia magna*, copper has some effect on the survival rate. *D. magna* was found dead after 24 h due to the accumulation of copper in the walls of the heart, which then blocked blood supply throughout the body [36]. This bradycardic effect has also been shown in other invertebrates, such as the blue mussel (*Mytilus edulis*) [37].

### 1.3. GHK and Copper Ion Complex Formation

The GHK tripeptide has also been recognized as a metal ion carrier in biological fluids [38], where it synergistically forms a complex with copper ions, which enhances its uptake into cells [2,38,39,40,41]. This complex causes the redox activity of Cu(II) silencing, allowing the delivery of nontoxic copper in cells [42,43,44] and activating beneficial actions in organs and tissues, such as aiding skin, intestines, blood vessels, bone [21], skin repair, and wound healing. It also possesses positive antioxidant, anti-inflammatory, and antianxiety properties, as well as causing an increase in cellular stemness and angiogenesis actions [22,43,45,46,47]. Furthermore, since GHK–Cu supports cell growth, it was suggested that the binding of copper with GHK might assist with transporting copper for cellular functions in cells in a manner that is nontoxic to cells [39]. A prior study by Zimei et al. (2016) reported that GHK-Cu increased collagen and elastin production by HDFa cells, depending on the mRNA expression of their TIMP (tissue inhibitor of metalloproteinases) over MMP (matrix metallopeptidase) [48]. Additionally, GHK-Cu (1 to 10 nM) stimulated the synthesis and breakdown of collagen and glycosaminoglycans [49]. GHK tempers the activity of metalloproteinase and its inhibitors (TIMP1 and TIMP2), regulating skin remodeling and wound healing processes [50,51]. In addition, GHK-Cu complexes have been recognized as excellent cosmetically therapeutic agents that can prevent oxidative stress in human skin [20], promote skin wound healing in rats [52], and ameliorate lipopolysaccharide-induced acute lung injury in mice [2]. Furthermore, the decline of GHK concentration with age also reduces its health-promoting benefits for many tissues, such as chondrocytes, human fibroblasts, and liver cells, also affecting tissue regeneration, angiogenesis, and nerve outgrowth. Copper-binding GHK has biological actions that counter age-associated diseases and conditions [47]. The gradual death of neurons, resulting in a loss of brain function, such as cognition, age-related dysregulation of biochemical pathways, and alterations of gene expression, is poorly understood and current therapies are still ineffective. In rats, the use of GHK-Cu could protect lung tissue from induced acute lung injury, and suppress the infiltration of inflammatory cells into the lung [53]. However, the effect of GHK-Cu on cardiovascular function in zebrafish is, to date, unknown. Taken together, it is important to understand the working mechanisms and potentially beneficial effects of GHK-Cu complexes on protecting against cardiotoxicity, especially in zebrafish embryos, triggered by waterborne Cu exposure at different dosage concentrations and time periods, and even in various physiological environment conditions.

### 1.4. Zebrafish as an Animal Model to Study the GHK Tripeptide Effect on Chelating Copper Toxicity

An in vivo study in model organisms is important for learning and exploring the underlying mechanisms of toxicity, dysregulation, and bioavailability of copper in GHK-Cu form. In the present work, we use zebrafish as our model organism, which have a similar homology to humans [54,55]. Zebrafish are used as a basis for screening physiological effects of different metals, nanomaterials, and drug toxicities because of their excellent qualities of optical transparency at the embryonic stage, high fecundity, small size, cost-effectiveness, and rapid developmental process [56,57,58]. GHK has a high affinity for copper and copper ions, which in isolation are extremely toxic to aquatic animals. We, therefore, hypothesized that the administration of GHK tripeptides to zebrafish embryos may mitigate copper toxicity. To validate this hypothesis, we first performed recombinant GHK tripeptide expression and purification analysis and then performed physiological assessments on cardiotoxicity rescue testing with zebrafish embryos.

## 2. Materials and Methods

### 2.1. Plasmid Construction

pGEX-4T-1 was modified with a 6XHis tag in front of a GST tag at the N-terminus, and the thrombin protease cleave sequence was replaced by the TEV protease cleave site by a one-step site-directed deletion and insertion method [59]. The pGEX-4T-1 GHK construct was generated using a one-step site-directed deletion and insertion method [59] and was confirmed by DNA sequencing. The gene encoding the 6XHis tag TEV protease was synthesized and cloned into the pET28a (+) vector with an N-terminal tag by *Bam*HI and *Xho*I restriction enzymes. Here, *E. coli* BL21 (DE3) was used as the expression host, and both constructs were transformed by a heat shock method.

### 2.2. Protein Expression in E. coli

A 5-L vessel fermenter (Major Science Inc., Saratoga, CA, USA) was used in the cell culture experiments. The bacteria were grown in Luria-Bertani (LB) media in the presence of ampicillin (100 μg/mL) and chloramphenicol (34 μg/mL) at 37 °C until the cell density reached an OD_600_ of 0.6–0.8. The cultures were induced with 0.5 mM isopropyl β-d-thiogalactopyranoside (IPTG) for 3 h at 37 °C. The cells were harvested by centrifugation at 6000 rpm for 30 min at 4 °C, followed by snap-freezing in liquid nitrogen and storage at −80 °C.

### 2.3. Protein Purification by HisTrip Column and 6XHis Tag TEV Cleavage

Frozen bacterial pellets were resuspended in the lysis buffer (50 mM Tris-HCl, 150 mM NaCl, pH 8.0), and the cells were lysed using an ultrasonic processor. The cell lysate was then subjected to centrifugation at 27,000× *g* for 40 min, followed by ultracentrifugation at 220,000× *g* for 1 h at 4 °C to remove the cell debris and the membrane fraction from the lysis solution. Subsequently, we collected the supernatant solution after further purification by passing the lysis solution through a 0.22-μm filter. To obtain the purified recombinant GHK peptide, the crude protein was incubated with a HisTrip HP His tag protein purification column (GE Inc., Marlborough, MA, USA) for 2 h in a 4 °C room. After washing the column with the lysis buffer, TEV protease was added into the column for 1 h at room temperature to remove the GHK peptide from the HisTrip beads. The recombinant GHK peptide was then eluted with an elution buffer (50 mM Tris-HCl, 80 mM imidazole, pH 8.0). Finally, the eluted samples were concentrated and analyzed using SDS-PAGE and UV-vis spectroscopy.

### 2.4. SDS-PAGE

Electrophoresis was performed on a 13.3% acrylamide-SDS gel covered with a 4.5% acrylamide stacking gel. Both the 4.5% acrylamide stacking gel and the 13.3% acrylamide-SDS gel were prepared using a stock solution containing 38.67% (*wt*/*wt*) acrylamide and 1.33% (*wt*/*wt*) *N*,*N*-bis-(methylene acrylamide). Electrophoresis was carried out at 100 volts until the bromophenol blue marker reached the bottom of the gel (about 2.5 h). The gel was stained in a 0.25% (*wt*/*vol*) Coomassie Brilliant Blue G-250 solution containing 25% methanol for 20 to 30 min and then destained in a destaining solution containing 25% methanol and 7% acetic acid.

### 2.5. UV-vis Spectra

UV-vis spectra were recorded at a 1-nm resolution with a pair of quartz cuvettes with a Hitachi U2900 UV-vis double beam spectrophotometer. Different concentrations of the CuSO_4_ solution were prepared by mixing 0, 0.1, 0.2, 0.3, 0.4, 0.5, 0.6, 0.7, 0.8, 0.9, 1.0, 1.1, 1.2, and 1.3 equivalents of recombinant GHK peptides to a final volume of 1 mL. The samples were transferred to a quartz cuvette (1 cm path-length) for UV-vis absorption measurement. The UV-vis spectra of the copper titrations of the purified GHK are summarized in Figure 2C. No UV-vis absorption features were discernible for the copper-free recombinant GHK peptide. Upon adding CuSO_4_, the recombinant GHK peptide was converted to a GHK-copper complex, with the characteristic ligand-to-metal charge transfer bands at 246 nm.

### 2.6. X-Ray Absorption Spectroscopy

The X-ray absorption spectroscopy data were collected at the National Synchrotron Radiation Research Center (NSRRC; 17C wiggler beamline, Si (111) double crystal monochromator) in Hsinchu, Taiwan. The preparation method of the X-ray absorption experimental solution sample was to mix the equivalent molar stoichiometry of recombinant GHK and copper sulfate in a Tris-HCl buffer (50 mM, pH 7.4). All samples were loaded into a sample holder (1.4 × 1.4 × 0.2 cm) covered with sheets of Kapton. During the measurements, the samples were maintained at room temperature. Fluorescence data were collected using a solid-state detector equipped with a Ni filter and Soller slits. The data represent an average of 9 scans. Data reduction included energy calibration by assigning the first inflection point of Cu foil to 8980.3 eV. All of the data collection and processing were carried out using the ATHENA software package.

### 2.7. Determine the Concentrations and Molecular Weight of Recombinant GHK Peptide by HPLC

The retention time and concentrations of the recombinant GHK peptide and GHK standard (Sigma-Aldrich, Darmstadt, Germany) were determined by reverse-phase (Mightysil, RP-18, GP 250-4.6, 5 mm, Kanto Chemical Co., Inc., Tokyo, Japan) high-pressure liquid chromatography (Hitachi High-Performance Liquid Chromatograph Chromaster, Hitachi High-Technologies Corporation, Tokyo, Japan) analysis. Prior to each run, the column was pre-equilibrated in 20% acetonitrile in deionized water. A constant gradient of 20% acetonitrile was applied at a flow rate of 0.5 mL/min over a 10 min elution. The integrated GHK standards (0.1, 0.2, 0.5, 0.75, 1.0, and 2.0 mg/mL) and recombinant GHK peptide peak areas (l = 254 nm) for the different time points were used to monitor the changes in concentration over time. To determine the molecular weight, the recombinant GHK peptides were dissolved in a 50 mM pH 7.4 Tris-HCl buffer at room temperature.

### 2.8. Zebrafish Maintenance and Ethics

The zebrafish care and maintenance system, breeding, and larvae-raising procedures employed in this study were in accordance with standard protocols [55]. Wild-type AB strain zebrafish were used for breeding and observation in this study. All experimental protocols involving zebrafish were approved by the Institutional Animal Care and Use Committee of Chung Yuan Christian University with the approval number 10,825 (25 December 2019).

### 2.9. Zebrafish Acute Toxicity Test

The lethality tests of the compounds tested were conducted with 24 larvae in a 9-cm Petri dish for four days. Embryos were observed at 24 hpf for selection, where only the healthy embryos were used for further experiments. After collecting the embryos, copper sulfate pentahydrate and Gly-His-Lys acetate salt treatment of different dosages (1, 10, 100, 1000 nM) were added from 24 hpf until 96 hpf in separate dishes. The survival rates of each treatment were recorded every 24 h, and the compound solutions were renewed at the same time. Lethal concentrations were analyzed at the end of the recording, where nonlinear regression was used to evaluate a standard curve to show the lethality ratio.

### 2.10. Zebrafish Cardiac Performance Assay

Normally developed embryos conserved in 9-cm Petri dishes were selected at 24 hpf, and treatments followed for 48 h with various concentrations of compounds. Larvae at 72 hpf were set in 3% methylcellulose to keep them stable during recording with a high-speed digital charged coupling device (CCD; AZ Instrument, Taichung City, Taiwan) constructed with an inverted microscope (ICX, Sunny Optical Technology, Zhejiang, China) using a Hoffman 40× objective lens to capture high-quality video. The HiBest Viewer (AZ Instrument, Taichung City, Taiwan) software package was employed for recording at a speed of 200 fps for 10 sec. The video recording was focused on the ventricle movement for the measurement of the time interval, heart rate, stroke volume, cardiac output, ejection fraction, and shortening fraction. Analysis of these endpoints was carried out using an ImageJ-based method (available online: https://imagej.net/Fiji/Downloads). Time interval analysis was done using the Time Series Analyzer V3 plug-in (available online: https://imagej.nih.gov/ij/plugins/time-series.html) for ImageJ to analyze the pattern of change in the dynamic pixel intensity over time. The OriginPro 2019 software package (Originlab Corporation, Northampton, MA, USA) was used to determine the time of each peak, and the peak analyzer function was used to determine the time interval of each peak. Heart rate was defined as beat per minute (BPM) and was calculated by dividing 60 with the time interval acquired from OriginPro 2019.

### 2.11. Zebrafish Cardiac Performance Endpoint Calculation

A Poincare plot was generated by plotting the time interval of each peak using the Poincare plot plug-in for OriginPro 2019 (available online: https://www.originlab.com/fileExchange/details.aspx?fid=404). The sd1 and sd2 values generated from the Poincare plot analysis were used as the endpoint for heart rate variability. Stroke volume was calculated by subtracting the volume of the heart chamber at the end of the diastolic phase with the volume at the end of the systolic phase at the ventricle, with the assumption of the heart chamber being ellipsoid in shape. Cardiac output was determined by multiplying the stroke volume with the heart rate at the ventricle. Ejection fraction was calculated by dividing the stroke volume with the heart volume at the end of the diastolic phase, and the data are shown here as percentages. The shortening fraction was determined by dividing the difference of the heart chamber width at the end of the diastolic phase and the end of the systolic phase with the width of the heart chamber at the end of the diastolic phase (Figure 3A–C).

### 2.12. Statistics

All statistical data were generated using the GraphPad Prism software package (GraphPad Software version 8 Inc., La Jolla, CA, USA). To determine the level of significance, parametric or nonparametric *t*-tests were performed, depending on the data distribution. The level of significance was set at a *p*-value < 0.05.

## 3. Results

### 3.1. Overview of Experimental Design and Workflow

**Figure 1:**
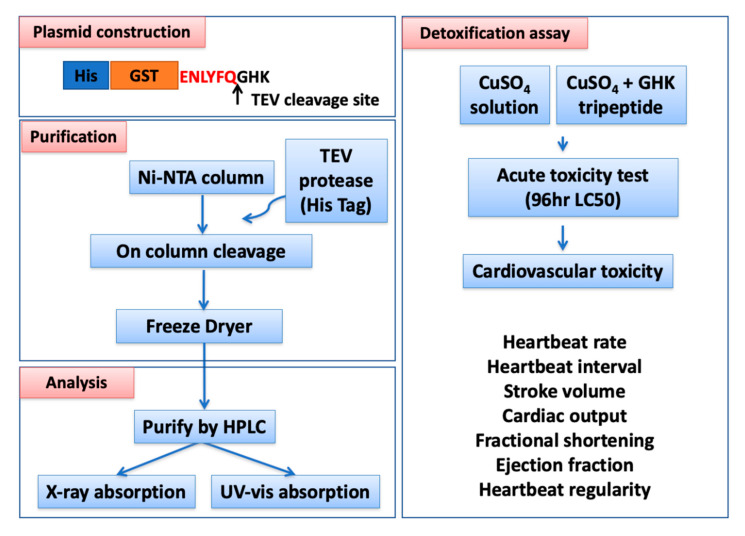
Overview of the experimental design and workflow, including four major parts, namely, plasmid construction, recombinant GHK (glycyl-histidyl-lysine) tripeptide purification, biochemical property analysis, and detoxification assays, in zebrafish embryos.

The specific aim of this study is to produce recombinant GHK tripeptides and elucidate whether recombinant GHK tripeptides are highly potent agents in protecting from copper cardiotoxicity in vivo. To reach this goal, an expressional plasmid was constructed, carrying a GHK tripeptide fused with histidine-glutathione-S-transferase (GST) double tags and tobacco etch virus (TEV) cleavage sites at the N-terminus. All of the experiments were performed under pH 7.4. We successfully achieved large-scale production of the recombinant GHK tripeptide in *E. coli.* via molecular cloning technology. After on-column TEV enzyme digestion, the C-terminus-tagged recombinant GHK tripeptide was released from the GST beads, and the eluted recombinant GHK tripeptide was immediately purified by high-performance liquid chromatography (HPLC). The purified recombinant GHK tripeptides, with/without a copper associate, were subjected to X-ray absorption spectroscopy and UV-vis absorption analysis for biophysical and biochemical characterization. Finally, we used zebrafish as an in vivo model to address the detoxification potential of recombinant GHK tripeptides by evaluating LC50 and cardiotoxicity (the experimental design and workflow are both summarized in Figure 1).

### 3.2. Expression, Purification, and Characterization of GHK Tripeptide

**Figure 2:**
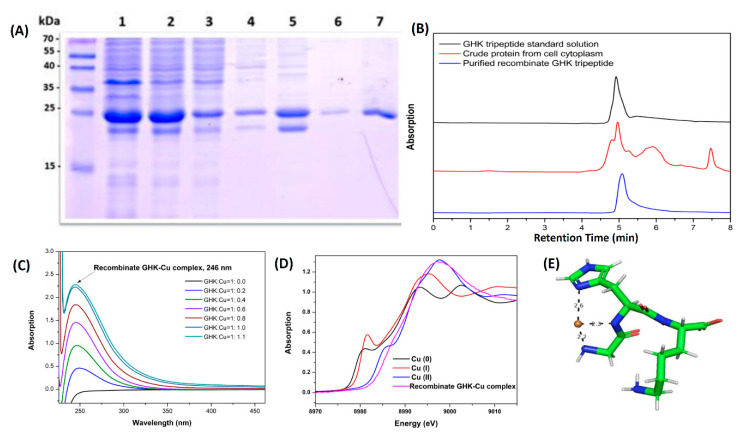
Expression, purification, and characterization of the recombinant GHK tripeptide. (**A**) Expression of a recombinant protein containing a GHK tripeptide. Lane 1: cell total lysate; Lane 2: crude protein in the supernatant part; Lane 3: flowthrough by a Ni-NTA (nitrilotriacetic acid) agarose base his-taq affinity column; Lane 4: elute with 0 mM of imidazole in a Ni-NTA agarose base his-taq affinity column; Lane 5: elute with 40 mM of imidazole in a Ni-NTA agarose base his-taq affinity column; Lane 6: elute with 80 mM of imidazole in a Ni-NTA agarose base his-taq affinity column, Lane 7: Ni-NTA agarose bead content with a recombinant 6XHis-GST-GHK peptide. (**B**) The recombinant GHK peptide fractions were purified with a C18 reverse-phase column by HPLC. Both of the retention times of recombinant GHK peptide (red color) and GHK standard (black color) were 5 min. (**C**) Spectrophotometry analysis of copper and recombinant GHK peptide interaction at different molar ratio combinations. The specific absorption peak of the recombinant GHK-Cu complex was 246 nm. The concentration of the recombinant GHK peptide was 1 mg/mL. (**D**) X-ray near-edge spectrophotometry analysis of copper foil (Cu(0)), Cu_2_O (Cu(I)), CuO (Cu(II)), and the recombinant GHK-Cu peptide. (**E**) X-ray crystal structure of the GHK peptide when associated with the copper ion.

To obtain sufficient supplies of the GHK peptide for the present study, the recombinant GHK peptide was cloned and overexpressed in *E. coli* BL21 (DE3) cells with 6XHis and GST affinity tags. In 5-L fermentation system runs, sufficient quantities of the purified protein were obtained for this study. From the fermentation system, we could harvest around 8–10 g of *E. coli* cells per liter of Luria-Bertani (LB) broth media, with around 50 g of *E. coli* cells per batch of cell culture. According to the SDS-PAGE analysis results from Figure 2A, the expression yields of recombinant GHK were around 50% in the crude protein from the cell lysate.

The recombinant GHK peptide was isolated from the cytoplasm of the host cells and purified as follows. The recombinant GHK-containing fractions were first collected by Äkta FPLC (fast performance liquid chromatography) via an immobilized metal affinity chromatography (IMAC) HisTrap HP His tag protein purification column. These recombinant GHK-peptide fractions were further purified by a C18 reverse column by HPLC to remove protein impurities and endotoxin.

The fraction of the highly purified recombinant 6XHis-GST-GHK peptide collected by a fraction collector was characterized by SDS-PAGE analysis. The highly purified recombinant 6XHis-GST-GHK peptide appeared as a polypeptide at 25 kDa with the SDS-PAGE analysis (Figure 2A, lane 7). The SDS-PAGE band at 25 kDa was subjected to on-column cleavage, and the peptides were purified by HPLC (Figure 2B). Depending on the quantitative analysis by HPLC (Figure 2B), the isolated and purified yields of recombinant GHK were estimated to be around 0.3 mg/g of *E. coli*.

According to the UV-vis absorption spectra of the recombinant GHK-Cu, since the copper-peptide complex formed by the copper ions and GHK peptides appeared as a d-d band transition feature, the signal of the characteristic absorption peak at a wavelength of 246 nm was observable in the UV-vis absorption spectrum. The recombinant GHK peptide chelates with a copper ion to form GHK-Cu, where the absorption intensity at 246 nm is continuously raised until the GHK:Cu ratio is 1:1, which means that the concentration of the copper-peptide complex has been saturated (Figure 2C).

X-ray absorption near-edge spectroscopy (XANES) is an important tool to monitor the oxidation state of copper ions in recombinant GHK-Cu peptides. The experiment results indicated that the Cu K-edge feature (1s to 4p) from the recombinant GHK peptide-associated copper showed significant edge shifts at 8979 and 8984 eV and overlapped the edge jump of the standard form of Cu(II) (CuO). According to the evidence from XANES, we confirmed that the valence state of the copper ion associated with the recombinant GHK was an oxidized form of Cu(II) (Figure 2D).

In addition to the copper-binding property, the molecular structure has previously been studied by X-ray absorption spectroscopy and X-ray crystallography [43]. The structure (Figure 2E) shows glycine amino nitrogen, the deprotonated amide nitrogen of the glycine-histidine peptide bond, and the nitrogen of the imidazole side chain of the histidine and a labile oxygen atom from a carboxylate ligand [43]. Those studies have demonstrated that GHK can interact with Cu(II). To investigate Cu(II) coordination in recombinant GHK, X-ray absorption spectra were analyzed via XANES. The XANES results for the recombinant GHK showed a specific spectral feature at 8988 eV (1s to 4p), which was in agreement with a previous report of the Cu(II) GHK peptide complex structure in an aqueous solution [55]. A square pyramidal structure was observed from the XANES signature peaks of copper ions, which are associated with recombinant GHK.

### 3.3. Bioassay of GHK Tripeptide on Protecting Copper Toxicity in Zebrafish

**Figure 3:**
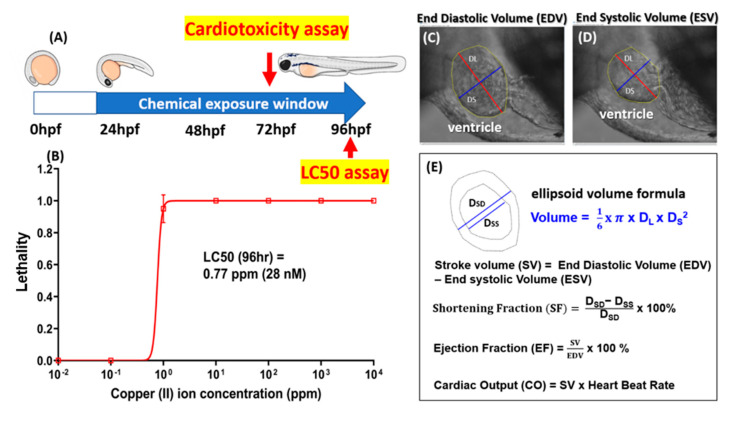
The experimental design, 96 h LC50, and mathematical formulae used to calculate cardiac performance in zebrafish. (**A**,**B**) Zebrafish embryos aged 24 h postfertilization (hpf) were treated with GHK tripeptide and copper combinations for 48 h continuously, and the cardiac-related endpoints, as well as mortality (LC50), were measured at 72 and 96 hpf, respectively. The LC50 values were predicted by a curve-fitting method. The outlooks of the ventricles were determined at either the diastolic (**C**) or systolic (**D**) phases. (**E**) The mathematical formulae used to measure the shortening fraction, ejection fraction, stroke volume, and cardiac output in the ventricle were investigated for zebrafish embryos aged 72 hpf.

Copper is relatively toxic to zebrafish and can induce abnormalities in embryonic development, deformation, and cardiac and locomotor dysregulation, even with exposure to a very low dose [35,60,61,62]. The potential rescue effect of a GHK tripeptide on protecting from cardiotoxicity triggered by waterborne copper exposure was examined in this study. Healthy zebrafish embryos aged 24 hpf were selected and subjected to waterborne copper incubation, either alone or with a GHK tripeptide. Zebrafish embryos were subjected to cardiac function assays by 72 hpf, and acute toxicity was measured by 96 hpf (Figure 3A). For the acute toxicity test, the 96 h LC50 level was measured and was predicted to be as low as 0.77 ppm (around 28 nM) by a curve-fitting method after the zebrafish embryos were treated with different doses of Cu^2+^, from 10^−2^ to 10^4^ ppm (Figure 3B).

**Figure 4:**
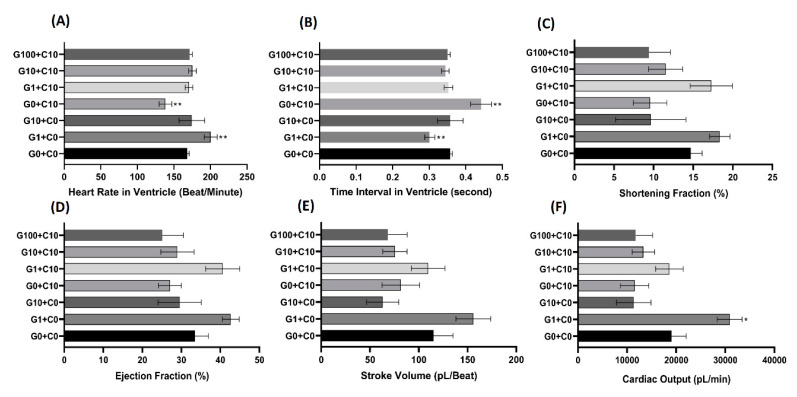
Results for heartbeat rate, heartbeat interval, shortening fraction, ejection fraction, stroke volume, and cardiac output in zebrafish embryos after treatment with GHK tripeptide and copper combinations. Zebrafish embryos aged 24 hpf were treated with GHK tripeptide and copper combinations for 48 h continuously, and the cardiac-related endpoints were measured: (**A**) heartbeat rate, (**B**) heartbeat interval, (**C**) shortening fraction, (**D**) ejection fraction, (**E**) stroke volume, and (**F**) cardiac output in the ventricle. G = GHK (nM), C = CuSO_4_ (nM). G0 + C0 was used as a control group. G1 + C0 and G10 + C0 were used as GHK function test groups. G0 + C10 was used as a cardiotoxicity group raised by copper exposure. G1 + C10, G10 + C10, and G100 + C10 were used as cardiotoxicity rescue groups. The significance was assayed by a one-way ANOVA with a nonparametric Mann-Whitney test (* *p* < 0.05, ** *p* < 0.01). In each tested group, the sample size was between 10 and 15.

For a cardiovascular performance assay, six endpoints were chosen to evaluate the cardiovascular performance in the zebrafish larvae: the heart rate and time interval in ventricles, stroke volume, cardiac output, ejection fraction, and shortening fraction. The calculation principles and mathematical formulae for these cardiophysiological parameters are summarized in Figure 3C–E. The use of a high-speed digital charged coupling device (CCD) and a Hoffman lens enabled the ventricle volume and other parameters to be measured with high precision in concordance with our previously published method [63]. A significant decrease in heart rate at the ventricle was observed after incubation at a sublethal dose of CuSO_4_ at 10 nM, but this bradycardia phenotype was rescued if the Cu^2+^-exposed embryos were cotreated with a recombinant GHK tripeptide, even at the lowest concentration of 1 nM. These results clearly demonstrated that the GHK peptide can minimize the adverse bradycardic effect caused by CuSO_4_, even at a 1:10 ratio (Figure 4A). A similar pattern was also shown for the time interval in the zebrafish ventricle (Figure 4B). A significant increase in the ventricle time interval was observed after incubation in 10 nM of CuSO_4_, while no significant change was observed if the CuSO_4_ was cotreated with a GHK peptide at every concentration (Figure 4B). In addition, it is intriguing to find that the administration of a GHK tripeptide alone, at a low dose (G1+C0), can boost the heartbeats of zebrafish embryos (Figure 4A).

The shortening fraction is the dimension of the heart chamber that is lost during the end-systolic phase and is often used to assess cardiac function during cardiotoxicity tests [64]. In this research, we observed a decrease of the shortening fraction after incubation in 10 nM of CuSO_4_ and with the cotreatment of 10 and 100 nM of the GHK peptide, although this was not statistically significant when compared to the control group (Figure 4C).

The ejection fraction is the fraction of blood volume ejected from a heart chamber with each contraction. The ejection fraction denotes the efficiency of a heart to pump blood [65]. In this study, we observed no significant decrease for the zebrafish ejection fraction after incubation in 10 nM CuSO_4_ and with cotreatment with 10 and 100 nM of the GHK peptide (Figure 4D).

Stroke volume is the volume of blood leaving the heart chamber after every contraction. In this study, the stroke volume was calculated by subtracting the heart volume at the end-diastolic phase by the end-systolic phase. The calculation of the heart volume was carried out by assuming that the heart chamber has an ellipsoid shape (Figure 3E). We observed no significant decrease after incubation in CuSO_4_ and after cotreatment with all the proposed concentrations of the GHK peptide. An increase in stroke volume was observed in the group incubated with 1 nM of the GHK peptide only, but this was not statistically significant compared to the control group (Figure 4E).

Cardiac output is the total volume of blood pumped from the ventricle per unit of time, which is calculated by multiplying the stroke volume with the heart rate. Cardiac output is related to the quantity of blood circulated to various parts of the body, so it reflects the efficiency of the heart to meet the body’s demands. In this study, we did not observe any significant change in cardiac output after incubation in CuSO_4_ or with cotreatment with the GHK peptide. It is intriguing to notice that incubation with 1 nM of GHK alone can significantly increase the cardiac output of zebrafish larvae, suggesting that the GHK tripeptide has a beneficial effect on cardiac output in zebrafish embryos (Figure 4F).

**Figure 5:**
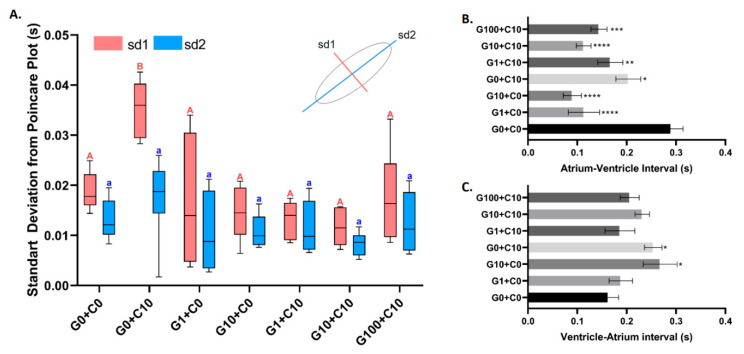
Sd1 and sd2 data generated from Poincare plot (**A**) and the time interval between the atrium and the ventricle chamber contractions, respectively (**B**,**C**), to compare the heartbeat variability in zebrafish embryos after being treated with different GHK tripeptide and copper combinations. The data were analyzed statistically using one-way ANOVA, with *p* < 0.05. Different labels above the shaded box indicate statistical significance. G = GHK (nM), C = CuSO_4_ (nM). G0 + C0 was used as a control group. G1 + C0 and G10 + C0 were used as GHK function test groups. G0 + C10 was used as a cardiotoxicity group raised by copper exposure. G1 + C10, G10 + C10, and G100 + C10 were used as cardiotoxicity rescue groups. In each tested group, the sample size was between 10 and 15.

A Poincare plot is a scatter graph that visualizes and quantifies the correlation and self-similarity between two consecutive data points in a time series. A Poincare plot was analyzed to check the regularity of each beat at the ventricle. In our research, we found that incubation with 10 nM of CuSO_4_ triggered the highest irregularity of heartbeat, which is shown by the higher sd1 value when compared with its control counterpart (Figure 5A). In line with our expectations, the cotreatment of CuSO_4_ with a GHK tripeptide at every concentration tested minimized the effect of CuSO_4_, which was shown by a similar value in both sd1 and sd2 (Figure 5A). This showed that the GHK tripeptide treatment was an effective method to rescue heartbeat irregularity induced by CuSO_4_ in zebrafish embryos. In addition, the biosafety of the GHK tripeptide is relatively high, since heartbeat regularity is maintained at the same level when compared to the control fish (Figure 5A).

We also investigated the regularity of the time interval between atrium to ventricle muscle relaxation and vice versa. Later, we found that every combination tested significantly decreased the interval time between atrium and ventricle chamber relaxation compared to the control (Figure 5B). A significant increase in the ventricle and atrium chamber time interval in the G10 + C0 and G0 + C10 combinations was also observed. However, even though the other combinations also increased the ventricle and atrium relaxation time interval, they were not significantly different from the control (Figure 5C). This showed that either GHK only, Cu only, or a combination of both GHK and Cu could alter the regularity of the atrium-ventricle and ventricle-atrium contraction time interval (Figure 5B,C).

## 4. Discussion

In this study, the potential protective effect of GHK-Cu complexes on cardiotoxicity in zebrafish embryos triggered by waterborne Cu exposure was investigated by performing the synthesis, characterization, and toxicity evaluation of GHK-Cu complexes. Results were obtained at different dosage concentrations and time periods, and in various physiological environment conditions. After the successful collection of a GHK tripeptide, its cardiotoxicity on normally developed zebrafish embryos at 24 hpf, for a subsequent 48 h incubation with only waterborne-Cu and Cu in a complex with the GHK peptide, was assessed. Cardiotoxicity was evaluated since many pathophysiological processes like tumor growth, oxidative stress, and cancer resistance contribute to inflammation, and cardiovascular and neurodegenerative diseases have been associated with copper imbalances [66,67].

Firstly, a recombinant GHK tripeptide was successfully cloned and overexpressed in *E. coli* as a fusion protein with histidine-GST double tags and TEV cleavage sites. Through rapid microorganism cultivation and simple liquid chromatography purification steps, a sufficient number of short peptides with biological activity and high economic value can be obtained. This recombinant protein expression approach can overcome the problem of limited access to GHK tripeptides due to their relatively high price. Moreover, this recombinant expression system was developed as an alternative to solid-phase peptide synthesis to reduce the use of hazardous chemicals. From the point of view of sustainable chemistry, a recombinant expression process reduces the negative impacts of chemical processes on human health and the environment (see Table A1 for the pros and cons of the two different approaches).

The copper reconstitution titrations of recombinant GHK tripeptide experiments were recorded by UV-vis absorption spectroscopy. The recombinant GHK revealed a strong association with copper, and the recombinant GHK to copper ratio was 1:1. X-ray K-edge absorption spectroscopy was used to monitor the oxidation level of copper in the recombinant GHK-Cu complexes. According to XANES, all of the copper ions were Cu(II). According to the above experimental data, the recombinant GHK produced by gene cloning technology exhibited the same biophysical and biochemical characteristics as the serum-isolated form of GHK.

In this study, six cardiophysiological endpoints were measured in zebrafish larvae to elucidate whether the cardiotoxicity raised by copper ions can be restored by GHK tripeptide administration as a result of its high affinity to bind copper. To the best of our knowledge, this is the first study on the effect of GHK-Cu on heart rate and other cardiovascular parameters. In this experiment, a significant decrease in heart rate and an increase in the heartbeat interval were observed in zebrafish embryos after incubation with 10 nM of CuSO_4_. However, this acute cardiotoxicity was rescued by GHK tripeptide administration, even at the lowest concentration of 1 nM, where the GHK-Cu complex was demonstrated to minimize the effects of CuSO_4_ toxicity at a GHK:Cu ratio of 1:10. These data showed that a GHK peptide could rescue cardiotoxicity induced by copper. In other cardiovascular function parameters, only the cardiac output showed a significant increase after exposure with 1 nM of the GHK peptide. Although there was an apparent reduction in the shortening and ejection fractions after incubation of the larvae with 10 nM CuSO_4_ by itself and in cotreatment with 10 and 100 nM of GHK peptide, this was not statistically significant when compared to the control group. A similar result was also observed for the stroke volume. Finally, the administration of GHK tripeptides could restore the heartbeat irregularity triggered by copper ion exposure.

Furthermore, the time interval between atrium and ventricle muscle relaxation can also be interpreted as preload time. If the preload time were to shorten, it would affect the amount of time taken for the ventricle chamber to load sufficient blood to pump to all parts of the body [68]. In this study, the incubation of zebrafish in GHK tripeptide and its combinations significantly increased the atrium-ventricle time interval. Previous studies on GHK have demonstrated that it can suppress fibrinogen, which plays a role in increasing blood viscosity by stimulating blood clot formation. This suppression results in a decrease in blood viscosity, which therefore decreases the resistance of blood entering the heart chamber and increases blood flow into the heart, which might be a possible mechanism of how GHK could decrease the time needed for the heart to preload the blood into the chamber [53].

In our previous survey, we noticed that several research papers have indicated that heavy metals complexed with EDTA (ethylenediaminetetraacetic acid) are biologically available and toxic. According to the literature of Guilhermino et al. (1997) [69], Cu(II)-EDTA complexes showed higher toxicity than their respective free metals in the acute toxicity test in the water flea *D. magna*. Tubbing et al. (1994) [70] also found that the photosynthesis of river microalgae was inhibited by Cu(II)-EDTA at low concentrations. Based on the scientific literature reports and considering the potential side effects of EDTA, we did not use EDTA as a material for control experiments in our experimental design.

In conclusion, the results collected from this study demonstrate that the copper treatment can induce heart rate irregularity in zebrafish embryos, and this adverse effect can be nicely rescued by administrating recombinant GHK tripeptide. For cardiophysiology, copper and GHK-Cu could only alter the heartbeat rate and regularity in zebrafish, without changing the dimension of the heart chamber. The data collected in our zebrafish study now paves the way for further testing of GHK tripeptide as a potent agent for detoxification or cardio-protection in rodents and other preclinical vertebrate animal tests.

## Appendix A

**Table A1:** Comparison of the pros and cons of the two major methods for GHK tripeptide production.

	GHK Tripeptide Production Methods	
Method	Recombinant expression (this study)	Solid-phase peptide synthesis (SPPS)
Productivity	Good reproducibility—recombinant GHK is overexpressed in *E. coli* successfully. High efficiency—large-scale and high-density *E. coli* cultures are achieved by a fermentation process. Simplifies purification by using one-step affinity column chromatography.	The SPPS method is mature—the porous solid resin matrix is stable and makes the reactants easily proceed in the voids. The polymerization of six amino acids can be completed in one day.
Sustainable	Eliminating the use of toxic chemical reagents and organic solvents.	Organic solvents like reprotoxic dimethylformamide are required.
Limitation	Cloning and molecular microbiology technology are required. Unable to prepare nonproteinogenic amino acids (e.g., D-form amino acids).	Length limited to 70 amino acid monomers or less. The price of peptide raw materials. Chemical synthesis yield.
